# Making Sense of Sex in Neuroscience

**DOI:** 10.1016/j.bpsgos.2024.100292

**Published:** 2024-03-15

**Authors:** Birgit Derntl, Steffen R. Hage, Manfred Hallschmid

**Affiliations:** a Department of Psychiatry and Psychotherapy, Women's Mental Health & Brain Function, Tübingen Center for Mental Health, University of Tübingen, Tübingen, Germany; b German Center for Mental Health, Tübingen, Germany; c Lead Graduate School, University of Tübingen, Tübingen, Germany; d Neurobiology of Social Communication, Department of Otolaryngology—Head and Neck Surgery, University of Tübingen, Tübingen, Germany; e Center for Integrative Neuroscience, University of Tübingen, Tübingen, Germany; f Department of Medical Psychology and Behavioural Neurobiology, University of Tübingen, Tübingen, Germany; g German Center for Diabetes Research, Tübingen, Germany; h Institute for Diabetes Research and Metabolic Diseases of the Helmholtz Center Munich at the University of Tübingen, Tübingen, Germany

The truth is rarely pure and never simple.

— Oscar Wilde

There seems to be a tacit agreement among many neuroscientists that biological sex is a negligible factor in the investigation of brain processes and function despite solid evidence to the contrary. Building upon previous commentaries, reviews (1,2), and calls to action (3), the recommendations put forward by Wierenga *et al.* (4) make the case for a concept of neuroscience that acknowledges and addresses the role of biological sex and the role of socially constructed gender. (As defined by the American Medical Association, “sex” is the classification of living things as male or female and is a biological component, defined via the genetic complement of chromosomes, including cellular and molecular differences. “Gender” comprises social, environmental, cultural, and behavioral factors and choices that influence a person’s self-identity and health.) The authors also provide hands-on suggestions that have the potential to transform current neuroscientific thinking and practice. Setting out with advice on how to appropriately assess sex (at birth and currently) and gender, the authors furthermore underline the fact that respective nonbinary differences need to be considered and outline guardrails for the adequate investigation of sex differences in terms of experimental design and statistical methods. To overcome the constraints of traditional group-based, case-control research, the authors argue that it should be combined with big data approaches to arrive at new insights while avoiding the pitfalls of the two individual strategies. Finally, their roadmap article calls on scientists to collaborate more closely, in the sense of participatory research, with (societal) stakeholders, especially with patients, families, and clinicians; this will necessitate sustained science communication efforts on the researchers’ part, which should be supported—in word and deed—by professional and funding bodies. Unfortunately, however, rampant sex omission and sex bias both in clinical trials (2) and in basic research (5) run counter to stipulations by key players such as the National Institutes of Health to consider sex (and gender) as essential variables.

SEE CORRESPONDING ARTICLE NO. 100283

Is it possible that the neuroscience community does not commit to the investigation of sex and gender differences because they seem to be first and foremost the domain of observational studies in humans? This notion is reflected in the current paper by the claim that animal models may not be a suitable means to grasp the complexity of this topic, although the authors explicitly acknowledge the importance of basic neuroscientific findings. There is of course a large toolkit of neuroscience methods for studying human brain structure and processes, for example the constantly refined techniques and analytical approaches in the field of neuroimaging as well as the broad spectrum of laboratory-based cause-and-effect experiments. Nevertheless, we would like to argue that animal models are essential for the detection and investigation of mechanisms that establish the impact of biological sex on brain function and neurobehavioral phenotypes. Moreover, mechanistic insights generated in animal research are urgently needed to bridge the gap between clinical observations—for example, of sex differences in neurological and mental disorders (1,2)—and translational efforts. Such research can unravel the neurophysiological basis of sex differences in highly evolved behavior such as social communication, although at first glance, its sophistication in humans may cast doubt on this assumption. Songbirds exhibit distinct sex differences in the usage and production of learned vocal communication signals known as birdsong, which result from sexually dimorphic brain anatomy and physiology (6). Sex-specific activation patterns of hypothalamic neural circuits give rise to differences in mating and aggressive behavior between female and male mice; estrogens act neonatally to masculinize these neural substrates, while testosterone later amplifies the intensity of male behavior (7). Findings such as these illuminate the usefulness of comparative approaches for the identification of brain mechanisms that underlie sex differences in social interactions at the genetic, molecular, cellular, and systems level. Because of their relevance for mental health and well-being and the clear evidence for sex differences in the prevalence, symptomatology, severity, and clinical course of related disorders, further worthwhile targets of research into sex-dependent brain processes are homeostatic processes including the control of eating behavior and metabolism, as well as memory, emotion, and stress regulation. For example, fear conditioning, which is a prominent paradigm in neurobiological research, has been explored primarily in male animals, which typically demonstrate freezing behavior; female animals seem to be inclined to exhibit active behavior instead of freezing, which indicates greater diversity in fear responses in animal models than was previously thought [cf. (2)]. Notwithstanding the greater complexity of human behavior, findings like these may inform and inspire new investigations into the relevance of biological sex for specific behavioral traits and, importantly, into underlying central nervous mechanisms.

Embracing the full complexity of brain differences between females and males, and between women and men, was a designated aim of the participants of the Lorentz workshop that spawned the present comprehensive guide (4). This endeavor necessitates the consideration of developmental aspects, spanning from prenatal influences through transitional periods, triggered or accompanied by endocrine changes, to age-related decline (1). While cohort studies are key for the identification of ontogenetic periods that are associated with increased vulnerability or resilience in terms of brain health, research in animals can encapsulate the complete life span or large parts thereof in controlled settings and permits the identification of driving forces also across generations. But what about research in animals to investigate gender, a construct that comprises sociocultural attitudes, behaviors, and identities that are related to but go beyond biological sex? It could be argued that it is too far-fetched an idea to resort to animal models in the investigation of gender in interaction with, and opposition to, biological sex, not least because the systematic assessment of gender expression, gender identity, and environmental influences on gender is in its infancy even in human studies. Identity and self-perception are indeed difficult to address in animals due to the lack of self-consciousness in classical model systems. At a more basic level, however, it is feasible to manipulate animals in a way such that they display behavior atypical of their biological sex and to subsequently study them in interaction with their conspecifics. Female canaries masculinized with testosterone exhibit male-phenotypical vocal behavior, and notably, their songs trigger sexual response behaviors in untreated female listeners (8). It will be exciting to explore how such reciprocal sexual behavior modulates brain processes and behavior of the masculinized female caller. Thus, studying the psychobiological underpinnings of the impact of the social environment and social interactions on overtly sex-related behavioral phenotypes in animals may contribute to our understanding of the role of gender in brain health. While such approaches may only be able to unravel particular aspects, we argue that beyond their scientific relevance, basic mechanistic insights may also help ease the tension that often characterizes and distorts the public discourse on gender.

Wierenga *et al.* (4) mention that precision medicine will benefit from the investigation of sex and gender in neuroscience and mental health research. Taking into account an individual’s specific makeup in terms of genetics, biomarkers, phenotype, and psychosocial characteristics, precision medicine targets the optimization of the effectiveness of disease prevention and treatment and at the same time the minimization of side effects in individuals who are less likely to respond to a certain therapy. The diagnosis and management of disorders, as well as gender equity in health care, will be promoted by decision making that is sex- and gender sensitive. To pursue the visionary aim of personalized medicine in brain-related health care, much more effort needs to be devoted to the role of sex and gender in disease etiology, diagnostics, and interventions, as well as with regard to the development of novel drugs and technologies; the recommendations outlined here (4) and in related publications (1, 2, 3) are an important step in this direction.

As evidenced by reports in traditional and online media, the role of sex and gender differences in brain function and mental health is a fixture of public discourse and controversies. Many people share an interest in sex and gender because these are important aspects of how they perceive themselves and others. There are a lot of “common sense” assumptions about sex, gender, and mental health that are not grounded in facts. Moreover, sex and gender bias in existing and newly obtained datasets can affect the use of artificial intelligence in current health care developments. In this context, Wierenga *et al.* (4) stress the importance of stakeholder involvement and science communication. Sharing the authors’ White, cisgender, European background, we agree that the cultural and social/societal setting needs to be addressed when sex and gender are being discussed. For example, the emergence of gender stereotypes during childhood varies markedly depending on cultural background (9), and across different cultures, sex and gender may be expected to play differently accentuated or even diverging roles in mental well-being and disorders (10). The inclusion of individuals from diverse cultural contexts and invested in different gender cultures and policies is therefore of great relevance for our research, whereas a purely Western perspective will significantly limit what we can achieve. As neuroscientists, we need to obtain reliable information about the many aspects of sex and gender differences and commonalities and identify their neurobiological bases to arrive at concepts that incorporate and respect the full spectrum of human diversity.
